# First Introduction of Syphilis into Scotland

**Published:** 1842-09

**Authors:** 


*First Introduction of Syphilis into Scotland.*—At the Medico-Chirurgical Society of Edinburgh, Professor Simpson read a communication relative to the history of the first introduction and diffusion of syphilis in Scotland at the end of the fifteenth century. He cited official edicts of the Aberdeen Magistrates, and of the king's privy council respecting the new 'infirmitez,' issued during the currency of 1497, with various casual notices of the distemper from the contemporaneous writings of Dunbar, Lyndsay, Inglis, &c. In these early times the disease passed in Scotland under the various names of grand gore, gorre, pockis, spanie pockis, French infirmitez, &c. Dr. S. alluded to an entry in 1502–3 in the privy purse expense book of Elizabeth, Queen of Henry VII., as containing the earliest notice of the disease in England, that he had been able to meet with. The first Scottish edicts were made with a view of arresting the progress of the disease, and ordained the application of a hot key of iron to the cheek, as the punishment of those who infringed their regulations. The Aberdeen edict was dated only four years and a month after the return of Columbus from his first voyage to Hispaniola. This edict was remarkable as being founded on the idea that the new malady disseminated itself by impure sexual intercourse—while, as is well known, it was not for several years afterwards, that this opinion of its mode of propagation, was adopted by the medical men and authors of these times. The Edinburgh regulations pre-supposed the disease to be communicable even by the persons that took the cure upon them, although these persons were themselves uninfected. Lastly, Dr. S. proceeded to show from the old data which he had adduced, that syphilis was new to this part of Europe, at the date of the edicts, that it was hence different on the one hand from gonorrhœa, and on the other from the leprosy of the middle ages, both of which affections were well known in this country before the era in question.—*N. Y. Lancet,* from *Lond. and Edinb. Monthly Jour. Med. Sci.*.

